# Efficacy and potential pharmacological mechanisms of total glucosides of paeony in treating ankylosing spondylitis in Asian populations: a meta-analysis, network pharmacology, and molecular dynamics simulation

**DOI:** 10.3389/fnut.2026.1738150

**Published:** 2026-03-27

**Authors:** Wenjun Mu, Jianhui Ma, Xudong Zhang, Jun Su, Xiang Wang, Cong Huang

**Affiliations:** 1 Guizhou University of Traditional Chinese Medicine, Guiyang, China; 2 Anshun Hospital of Traditional Chinese Medicine, Anshun, China

**Keywords:** ankylosing spondylitis, meta-analysis, molecular dynamics simulation, network pharmacology, total glucosides of paeony

## Abstract

**Background:**

Ankylosing spondylitis (AS) is a chronic autoimmune disorder, accompanied by a notably high disability rate and limited treatment options. White peony root is a classic nourishing Chinese medicinal herb with immune-modulating effects. Its glycoside components-Total glucosides of paeony (TGP), a natural compound, exhibits anti-inflammatory and immunomodulatory effects. However, little is currently known about the efficacy and safety of TGP therapy for treating AS.

**Purpose:**

This study aimed to provide evidence-based support for the efficacy and safety of TGP in treating ankylosing spondylitis and to explore the potential mechanisms underlying TGP therapy for this disease.

**Methods:**

A search strategy combining keywords and free-text terms was employed to retrieve studies from eight databases: CBM, CNKI, VIP, Wanfang, Embase, PubMed, Cochrane Library, and Web of Science. Statistical analysis was conducted using RevMan 5.4 software, with evidence quality assessed via the GRADE system. Additionally, network pharmacology, molecular docking, and molecular dynamics simulations (constant pressure for 100 ns) were utilized to further analyze the potential targets and pathways involved in TGP’s therapeutic effects on AS.

**Results:**

The meta-analysis ultimately included 28 studies involving 2,130 patients, with results indicating that the TGP combination therapy group significantly improved spinal function in AS patients, reduced inflammatory responses, enhanced quality of life, and played a crucial role in anti-inflammatory and immune modulation with favorable safety profiles. The therapeutic targets of TGP in AS include TLR4, NFKB1, HSP90AA1, HIF1A, MTOR, ITGB1, and CXCR4, with its mechanism of action linked to TLR and NF-κB pathways. Molecular docking and MD simulations revealed benzoylpaeoniflorin exhibits the strongest binding affinity, suggesting TGP may regulate AS pathogenesis by interacting with TLR4.

**Conclusion:**

TGP demonstrates favorable clinical efficacy in treating AS, with a multi-targeted intervention mechanism that holds therapeutic promise.

**Systematic review registration:**

The study was prospectively registered in PROSPERO (Registration No. CRD: 420251111863, URL: https://www.crd.york.ac.uk/PROSPERO/).

## 1 Introduction

Ankylosing spondylitis (AS) is a chronic autoimmune disorder that primarily affects the spine, sacroiliac joints, and peripheral joints (1). Pathological new bone formation is a hallmark of the disease. Syndesmophyte development at bone–cartilage junctions, together with ossification of spinal ligaments, leads to progressive spinal fusion, restricted mobility, and functional impairment. Over time, these processes culminate in spinal ankylosis, deformity, and joint destruction, resulting in severe disability and reduced quality of life. AS imposes substantial physical and psychological burdens on affected individuals. The pathophysiological essence of AS lies in chronic inflammation driven by aberrant immune activation. The immune system remains in a persistent “battle” state, releasing pro-inflammatory mediators such as tumor necrosis factor-*α* (TNF-α) and various interleukins. This sustained inflammatory response consumes significant metabolic energy—comparable to a prolonged low-grade fever—thereby elevating the basal metabolic rate. The ensuing cytokine storm not only damages articular and spinal tissues but also disrupts systemic metabolism, accelerating protein catabolism and altering lipid metabolism. These changes contribute to muscle wasting and body weight fluctuations. Furthermore, increased energy expenditure, coupled with decreased appetite caused by pain and medication side effects, often results in unexplained weight loss among AS patients. Although the exact pathogenesis of AS remains incompletely understood, it is generally accepted to involve a multifactorial interplay among genetic, immune, and environmental determinants (2). Recent research suggests that genetic susceptibility and environmental triggers interact through gut microbiota dysregulation to influence pathological new bone formation in AS (3). Modern medical management of AS primarily relies on nonsteroidal anti-inflammatory drugs (NSAIDs), disease-modifying antirheumatic drugs (DMARDs), and biologics to alleviate inflammation and slow aberrant bone formation before the disease advances. However, adverse events and drug tolerance often restrict their long-term application. Traditional Chinese medicine (TCM) has been employed for centuries in the treatment of AS, demonstrating clinical efficacy in reducing disease activity, improving spinal mobility, suppressing inflammation, and enhancing quality of life (4). Elucidating the mechanisms underlying TCM’s therapeutic effects could aid in establishing standardized, evidence-based treatment protocols. Within TCM, white peony root (*Paeonia lactiflora* Pall.) holds a prominent place in both medicinal and dietary applications. It is incorporated into soups, infusions, and congees for its blood-nourishing, liver-soothing, and analgesic properties. Examples include: White Peony Root Braised with Pork Ribs—used for blood deficiency syndromes to replenish and nourish blood. White Peony and Licorice Tea—relieves muscle spasms and smooth muscle pain (e.g., abdominal discomfort in irritable bowel syndrome). Four Substances Decoction—a classical hematopoietic formula comprising Angelica sinensis, Ligusticum chuanxiong, white peony root, and prepared Rehmannia root, commonly used as a base for tonic soups. Pharmacological studies demonstrate that white peony root promotes bone marrow hematopoiesis, modulates endocrine and immune functions, and alleviates inflammation-induced pain.

Total Glucosides of Paeony (TGP) represent the principal active components extracted from *Paeonia lactiflora* root slices, consisting mainly of paeoniflorin, paeoniflorin lactone, and paeoniflorin oxime. TGP exerts potent immunomodulatory and anti-inflammatory effects (5). By regulating both T cell–mediated cellular immunity and B cell–mediated humoral immunity, TGP has been applied clinically to treat autoimmune diseases such as AS, rheumatoid arthritis, systemic lupus erythematosus, and Sjögren’s syndrome (6). Recent studies have confirmed that TGP can effectively downregulate the levels of inflammatory cytokines in patients with AS and modulate the TLR4- and TLR5-mediated immune inflammatory responses, suggesting that it may hold unique therapeutic value in controlling disease activity and improving patient prognosis. Despite its long-standing clinical use in China, robust evidence-based validation for TGP therapy in AS remains limited. Two major challenges persist: Lack of standardized treatment frameworks. Current clinical practice is largely empirical, with dosing regimens not yet optimized for different disease stages. Insufficient high-quality clinical evidence. Most existing studies are small-scale, single-center trials with limited population diversity, reducing the representativeness and generalizability of findings. Moreover, although preliminary pharmacological studies have explored TGP’s therapeutic mechanisms in AS, its precise molecular actions remain incompletely characterized—particularly concerning its multi-target regulatory networks and associated signaling pathways. These gaps hinder the establishment of standardized guidelines and limit the broader clinical adoption of TGP in AS management.

Therefore, this study integrates systematic reviews, network pharmacology, molecular docking, and molecular dynamics simulations to elucidate the potential mechanisms of TGP in treating ankylosing spondylitis (AS). Molecular docking and molecular dynamics simulations are employed to further validate the stability and binding affinity of TGP’s active components to key therapeutic targets. Collectively, these approaches aim to provide robust scientific evidence for elucidating the therapeutic mechanisms of TGP in AS, thereby promoting the standardization of its clinical applications, optimizing treatment strategies, and fostering innovation in the integration of traditional Chinese medicine (TCM) with rheumatology and immunology.

## 2 Methods

### 2.1 Meta-analysis evaluating the efficacy of TGP for AS

This study followed the PRISMA guidelines for systematic reviews and was prospectively registered in PROSPERO (Registration No. CRD: 420251111863).

#### 2.1.1 Literature search strategy

A comprehensive search strategy combining subject headings and free-text keywords was employed across eight databases—CBM, CNKI, VIP, Wanfang, Embase, PubMed, Cochrane Library, and Web of Science—covering all records from database inception to August 17, 2025.

#### 2.1.2 Inclusion criteria

a Study population: Subjects meeting the established AS classification criteria.
b Study type: Eligible studies were randomized controlled trials (RCTs) evaluating TGP for the treatment of ankylosing spondylitis (AS), published in Chinese or English.
c Intervention measures: In the experimental arms, AS patients received either TGP monotherapy or TGP combined with conventional therapies such as oral DMARDs, biologics, or surgical interventions. Control groups received placebo or standard conventional oral DMARDs, biologics, and surgical interventions.
d Outcome measures: Primary and secondary outcomes included the TCM efficacy rate, VAS score, duration of morning stiffness, inflammatory markers (ESR, CRP), BASDAI, BASFI, Schober’s test, and chest wall mobility.

#### 2.1.3 Exclusion criteria

a Duplicate, incomplete, or inaccessible full-text studies.
b Studies with non-rigorous designs or inconsistent intervention/control protocols.
c Studies lacking clearly defined outcome measures.

#### 2.1.4 Data extraction and quality assessment

Two independent reviewers performed literature screening, data extraction, and cross-validation. Discrepancies were resolved by discussion or adjudication by a third reviewer.

Extracted data included: first author, publication year, study location, study type, sample size, intervention details, relevant outcomes, and risk of bias parameters. Risk of bias was evaluated using the Cochrane Risk of Bias Tool. Evidence quality was assessed following the GRADE framework (7) with GRADEprofiler 3.6, considering five downgrading factors (risk of bias, inconsistency, indirectness, imprecision, publication bias) and three upgrading factors (large effect size, confounding minimization, dose–response relationship). The overall evidence quality was categorized as high, moderate, low, or very low (8).

#### 2.1.5 Statistical analysis

Statistical analyses were conducted using RevMan 5.4 software. For dichotomous variables, the relative risk (RR) was used as the effect measure, and 95% confidence intervals (CI) were calculated for each effect size. Heterogeneity among studies was assessed using the χ^2^ test (*α* = 0.1) and quantified by the I^2^ statistic. A fixed-effect model was applied when no significant heterogeneity was detected. When heterogeneity was present, its sources were investigated, and after excluding clinically evident heterogeneity, a random-effects model was used. The overall meta-analysis was performed at a significance level of *α* = 0.05. Obvious clinical heterogeneity was handled using subgroup, sensitivity, or descriptive analyses.

### 2.2 Study on the potential effects of TGP on AS based on network pharmacology, molecular docking, and molecular dynamics prediction

#### 2.2.1 Active component, target screening, and network construction of TGP for treating AS

Compounds related to total glucosides of paeony (TGP) were retrieved from the Traditional Chinese Medicine System Pharmacology Database and Analysis Platform (TCMSP)^1^ using the keyword “total glucosides of paeony.” Candidates with drug-likeness (DL) ≥ 0.18 and oral bioavailability (OB) ≥ 30% were selected. To avoid omitting potentially active compounds, those not meeting these thresholds were retained if animal studies provided clear evidence of anti-AS activity.

All AS-related targets were identified from GeneCards, TTD, and the Pharmacogenetics and Pharmacogenomics Knowledge Base (PharmGKB)^2^ using “Ankylosing Spondylitis” as the search term. Identified targets were merged and deduplicated using the Venn package in R 4.2.2. Both TGP-related and AS-related targets were imported into R to construct a Venn diagram, identifying intersection targets shared by the drug and the disease.

A drug–active ingredient–potential target network was then generated using Cytoscape 3.9.1. The Network Analyzer tool calculated degree values and betweenness centrality to identify key nodes, thereby determining the core bioactive components of *Paeonia lactiflora* in AS treatment.

In the STRING database,^3^ the “Multiple Proteins” option was used with “*Homo sapiens*” as the species. Intersection targets were uploaded with a minimum interaction score of 0.4, and free nodes were hidden. The interaction results were exported as a TSV file, imported into Cytoscape 3.9.1, and analyzed with the CytoNCA plugin to compute Degree Centrality (DC) values. Nodes in the PPI network were visually scaled and colored according to their DC values to highlight core targets.

Using R 4.2.2, the following packages were installed: colorspace, stringi, ggplot2, DOSE, clusterProfiler, enrichplot, and pathview. GO functional enrichment and KEGG pathway analyses were performed on the identified potential targets. Results were ranked by ascending *p*-value, and the top 10 GO terms and top 30 KEGG pathways were visualized for each functional category.

#### 2.2.2 Molecular docking

The 2D structures of small-molecule ligands were obtained from PubChem^4^ and converted to 3D structures using ChemOffice, saved as .mol2 files. Corresponding protein targets were retrieved from the RCSB PDB database,^5^ selecting crystal structures with higher resolution as receptor models. Protein preprocessing, including dehydration and dephosphorylation, was performed using PyMOL 2.6, and the resulting structures were saved as PDB files. Both proteins and ligands were prepared in AutoDock 1.5.6, where hydrogenation and torsion determination were conducted, followed by definition of the docking box coordinates. AutoDock Vina was then employed to perform molecular docking and evaluate protein–ligand binding interactions. The docking conformation with the lowest binding energy was selected as the optimal complex. Visualization of 2D and 3D interactions was performed using Discovery Studio 2019 and PyMOL 2.6. Generally, binding energies < −5.0 kcal/mol indicate moderate affinity, whereas values < −7.0 kcal/mol suggest strong binding affinity. Lower binding energies correspond to stronger interactions, higher stability, and greater affinity.

#### 2.2.3 Molecular dynamics simulation

GROMACS 2022 was used to conduct molecular dynamics (MD) simulations. Force field parameters were generated using GROMACS pdb2gmx and the AutoFF web server. The amber14sb force field was applied to receptor proteins and the GAFF2 force field to ligands. The system was solvated within a 1 nm TIP3P cubic water box (9), and counterions were added using gmx genion to maintain electrostatic neutrality. Long-range electrostatics were computed via the Particle Mesh Ewald (PME) method with a 1 nm cutoff distance, and all bond constraints were enforced using the SHAKE algorithm. The MD simulations used a 1 fs integration step and the Verlet leapfrog algorithm. Before simulation, energy minimization was performed in three stages: (1) constraining solute molecules and minimizing water; (2) constraining counterions and minimizing energy; and (3) minimizing the entire system without constraints. Each stage comprised 3,000 steps of steepest descent followed by 2000 steps of conjugate gradient optimization. The system was then equilibrated under NPT conditions at 310 K and constant pressure for 100 ns. During the simulation, the g-RMSD, g-RMSF, g-HBonds, g-Rg, and g-SASA tools were employed to calculate root mean square deviation (RMSD), root mean square fluctuation (RMSF), hydrogen bonds (HBonds), radius of gyration (Rg), and solvent-accessible surface area (SASA), respectively.

## 3 Results

### 3.1 Meta-analysis results

#### 3.1.1 Literature search results, basic characteristics, and literature quality

**Figure 1:**
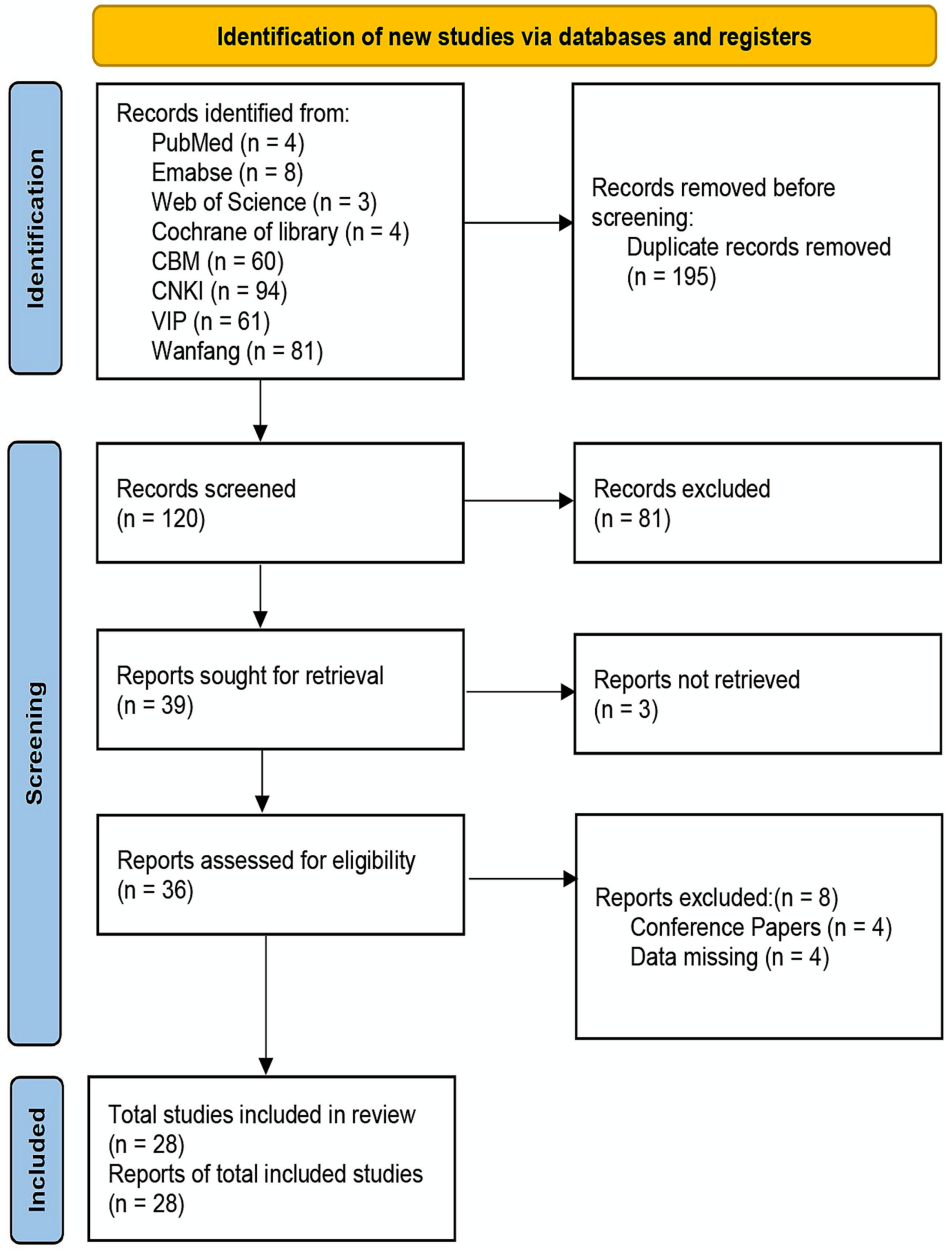
Literature search and screening process.

**Figure 2:**
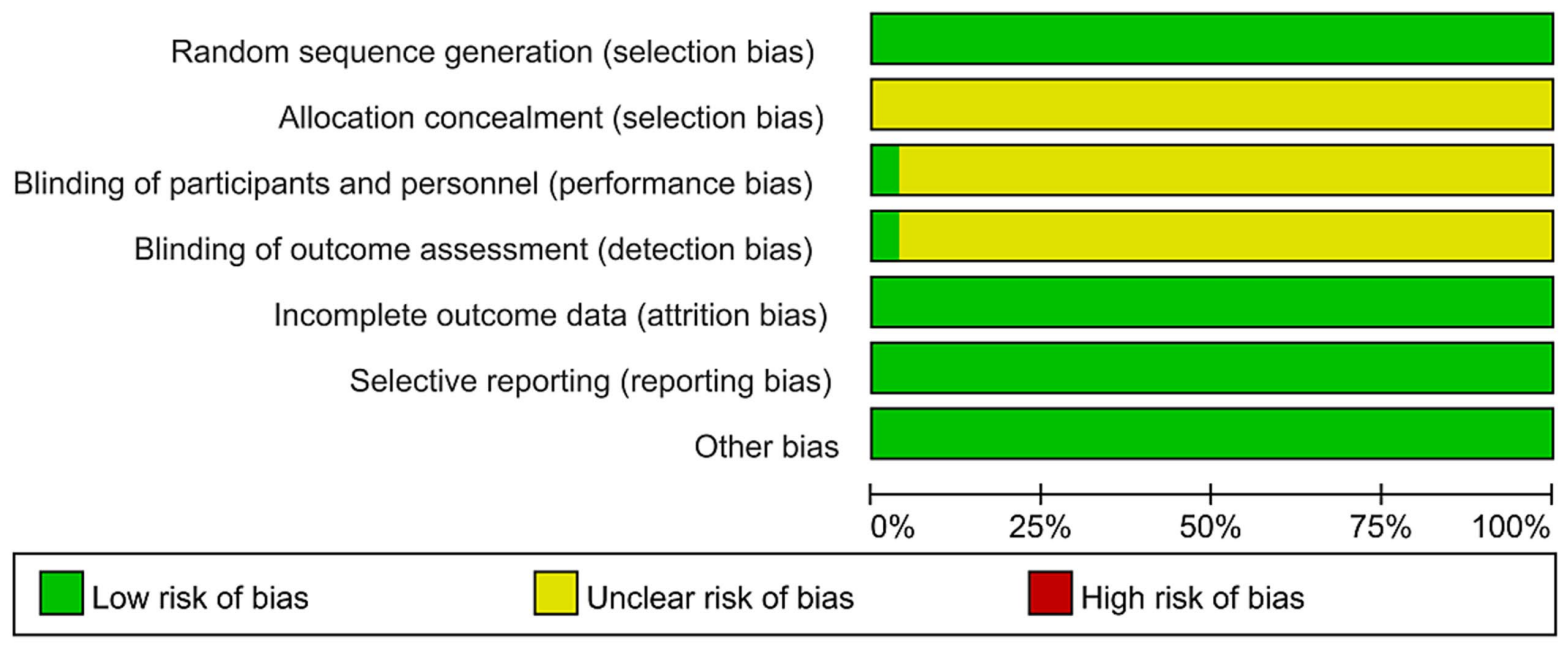
Methodological quality assessment.

Using a keyword-plus–free-text strategy, 315 relevant studies were initially retrieved from eight databases: CBM, CNKI, VIP, Wanfang, PubMed, Embase, Web of Science, and the Cochrane Library. After removal of duplicates, 120 unique records remained. Excluding non-RCTs, reviews, inconsistent research content, and systematic reviews yielded 39 studies. Among these, 3 studies were excluded due to unavailability of full-text articles. Following full-text assessment, 8 studies failed to meet inclusion criteria, resulting in a final dataset of 28 RCTs (10–38). The literature screening process is illustrated in Figure 1, the characteristics of included studies are summarized in Table 1, and the methodological quality assessment results are presented in Figure 2.

**Table 1:** Basic characteristics of the included studies.

Author	Year	T/C	Intervention measures		Treatment course (months)	Outcome indicator
			T	C		
Wang Yan	2008	29/26	TGP + SASP	SASP	6	①②③⑤⑥⑦⑩
Wang Suoliang	2007	34/33	TGP + SASP	MTX + SASP	6	①②③⑤⑥⑧⑨⑩
Li Chunxian	2006	50/30	TGP + SASP+NSAID	SASP+NSAID	6	⑦
Chen Yong	2004	24/27	TGP + SASP	SASP	6	①③④⑦⑩
Xiong Jinhe	2009	29/29	TGP + SASP+MTX	SASP+MTX	3	①②③⑤⑥⑩
Mou Tian	2017	36/36	TGP + SASP+MTX	SASP+MTX	3	①②③⑤⑥⑩
Xia Xuan	2010	22/20	TGP + SASP	SASP	6	①②③⑤⑥⑩
Su Lifang	2012	21/20	TGP + SASP	SASP	6	①②③⑤⑥⑦⑩
Zou Shiyong	2012	60/60	TGP + SASP	SASP	6	①②
Wu Jian	2014	40/40	TGP + Thalidomide	Thalidomide	3	①②③④⑧⑨⑩
Ruan Liyun	2013	40/40	TGP + MTX + Thalidomide	MTX + Thalidomide	6	①②③⑤⑩
Zhang Lu	2017	75/75	TGP + Thalidomide	Thalidomide	6	①②③⑤
Deng Zhaozhi	2005	40/40	TGP + SASP+MTX	SASP+MTX	3	①②④⑤⑥⑧⑨⑩
Pei Xun	2022	60/60	TGP + Diclofenac	SASP+Diclofenac	3	①②⑩
Li Wensi	2014	15/15	TGP + NSAIDs	NSAIDs	6	①②④⑧⑨⑩
Wan Yan	2009	30/25	TGP	SASP	6	⑦⑩
Liu Weili	2015	53/53	TGP + Diclofenac	SASP+Diclofenac	3	①②③④⑦⑧⑨⑩
Li Zhijun	2009	49/49	TGP	SASP	3	①②③④⑦⑧⑨⑩
Cao Senlin	2019	55/55	TGP + osteotomy	osteotomy	3	①⑦⑨⑩
Jiang Qiong	2012	34/34	TGP + LEF	LEF	12	①④⑧⑩
Zheng Hongxia (1)	2018	23/23	TGP + SASP+TG	SASP+TG	6	②⑩
Zheng Hongxia (2)	2018	24/25	TGP + SASP	SASP	6	②⑩
Liu Hui	2004	49/49	TGP + SASP	SASP	6	①③⑦⑩
Zhang Yu	2007	26/28	TGP + MTX + SASP	MTX + SASP	12	①②③⑤⑥⑦⑩
Chang Suguo	2013	19/19	TGP + SASP	SASP	6	⑦
Zhang Yabin	2015	44/44	TGP + MTX + Nimesulide	MTX + Nimesulide	3	⑦⑩
Wang Ying	2013	30/30	TGP + SASP	SASP	1	⑦
Liu Shuyu	2020	39/39	TGP + SASP+Thalidomide	SASP+Thalidomide	3	①②
Wang Zaihong	2012	28/28	TGP + SASP+NSAID	SASP+NSAID	6	①②③④⑦⑩

T, Treament group; C, Control group; SASP, Sulfasalazine; MTX, Methotrexate; NSAID, Nonsteroidal Antiinflammatory Drugs; TG, Tripterygium Glycosides; LEF, Leflunomide; ①ESR; ②CRP; ③Morning stiffness duration; ④VAS score; ⑤Schober; ⑥Chest expansion; ⑦TCM efficacy rate; ⑧BASDAI; ⑨BASFI; ⑩Adverse reactions.

#### 3.1.2 TCM efficacy rate

**Figure 3:**
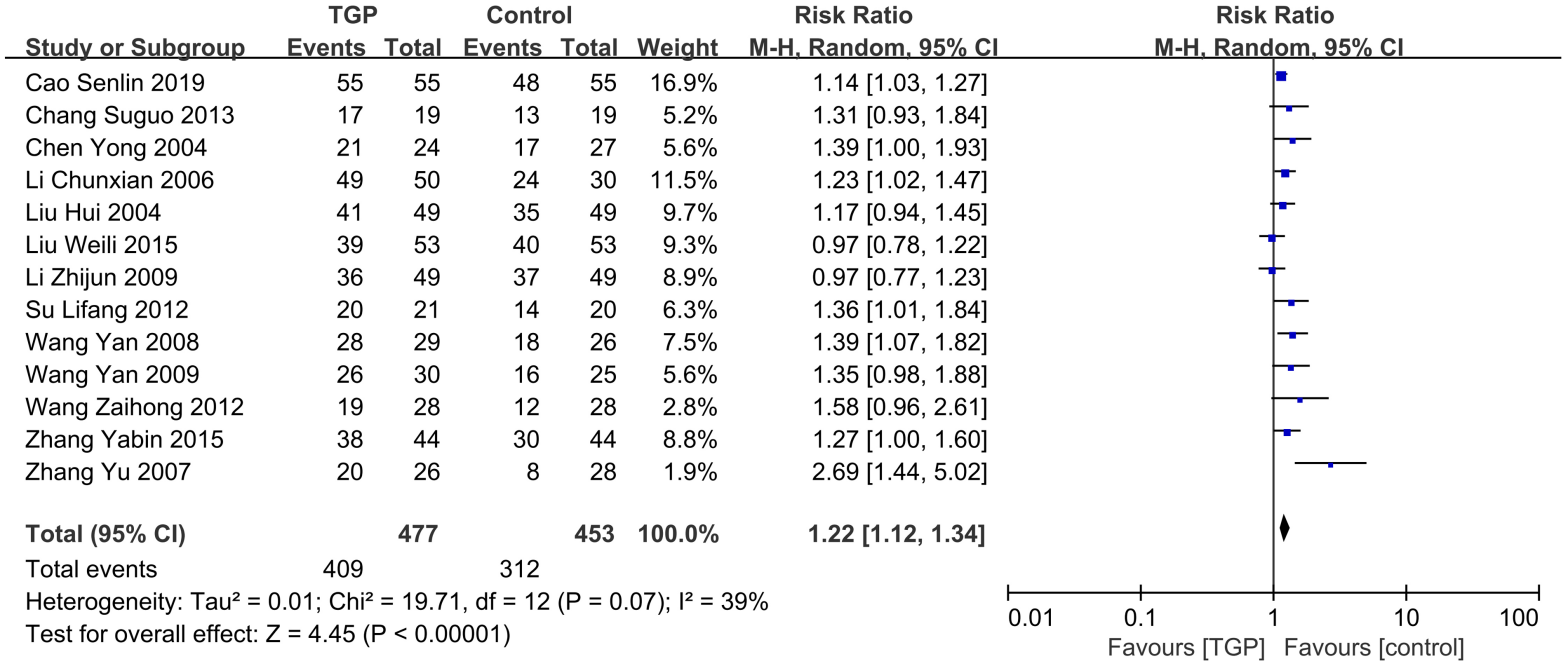
Forest plot of the association between TCM efficacy rate and AS.

A total of 13 RCTs (10, 11, 13, 15–18, 22, 23, 25, 30, 31, 35) involving 930 patients were included. Meta-analysis results indicated that TGP treatment achieved a significantly higher TCM efficacy rate in AS patients than the control group [RR = 1.22, 95% CI (1.12, 1.34), *p* < 0.00001]. The low heterogeneity (*I^2^* < 50%) reflected strong consistency among the included studies (Figure 3).

#### 3.1.3 VAS score

**Figure 4:**
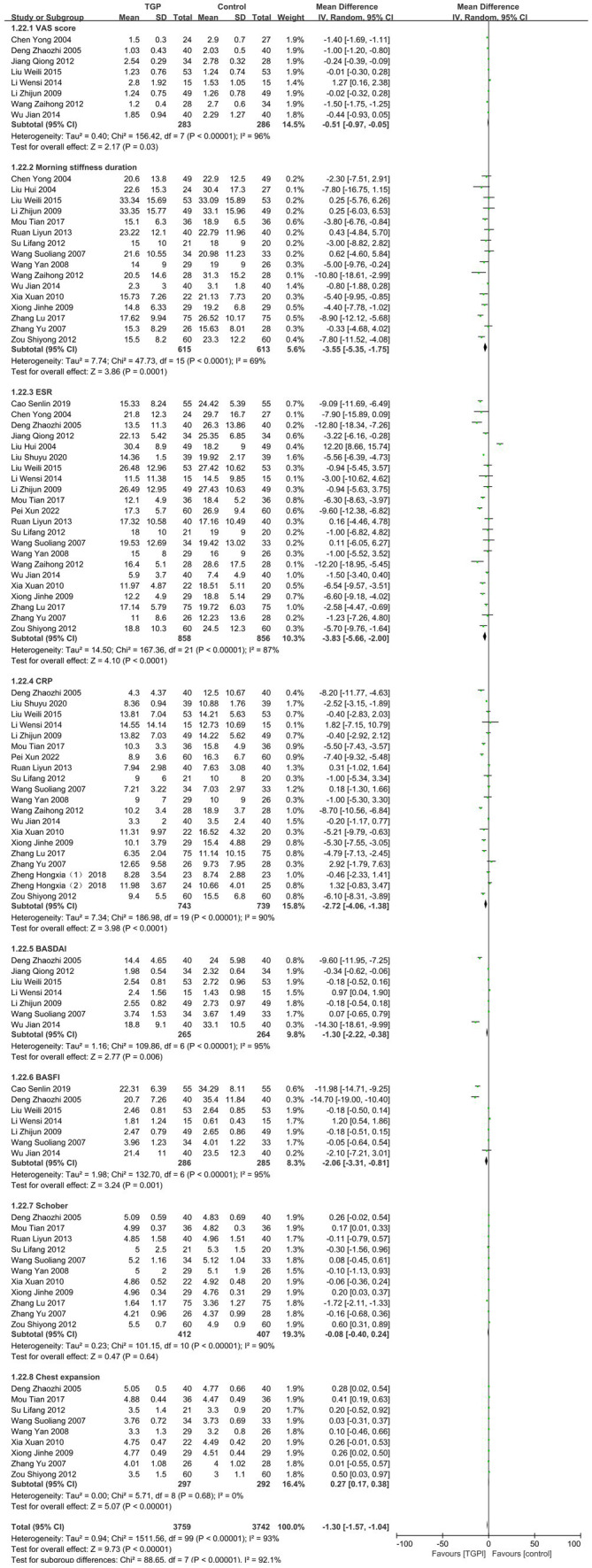
Forest plot of therapeutic efficacy in relation to AS.

A total of 8 RCTs (10, 12, 17, 21, 23, 28–30) with 569 patients were included. Meta-analysis revealed that the post-treatment VAS score was slightly lower in the TGP group compared to the control group [WMD = −0.51, 95% CI (−0.97, −0.05), *p* = 0.03]. However, the extremely high heterogeneity (*I^2^* > 90%) suggested substantial methodological variation among studies (Figure 4).

#### 3.1.4 Morning stiffness duration

A total of 16 RCTs (10, 11, 14–17, 19, 20, 22–24, 26, 29, 30, 32, 33) involving 1,228 patients were analyzed. Results demonstrated that TGP significantly reduced morning stiffness duration compared to the control group [WMD = −3.55, 95% CI (−5.35, −1.75), *p* = 0.0001]. Despite the statistical significance, high heterogeneity warrants cautious clinical interpretation of these findings (Figure 4).

#### 3.1.5 ESR

A total of 22 RCTs (10–12, 14–17, 19–24, 26, 28–30, 32, 33, 35, 37) involving 1,714 patients were included. Due to considerable heterogeneity (*I^2^* = 87%, *p* < 0.0001), a random-effects model was applied. Meta-analysis showed that post-treatment ESR levels were significantly lower in the TGP group than in the control group [WMD = −3.83, 95% CI (−5.66, −2.00), *p* < 0.00001; Figure 4].

#### 3.1.6 CRP

A total of 20 RCTs (12, 14, 17, 19, 20, 22–24, 26, 28–30, 32–37) involving 1,482 patients were analyzed. The TGP group demonstrated significantly lower CRP levels than the control group [WMD = −2.72, 95% CI (−4.06, −1.38), *p* < 0.00001]. Although statistically significant, the interpretation should consider the influence of extreme heterogeneity (*I^2^* = 90%; Figure 4).

#### 3.1.7 BASDAI

A total of 7 RCTs (12, 13, 17, 21, 28–30) involving 529 patients were included. Meta-analysis indicated that TGP significantly improved BASDAI scores compared with the control group [WMD = −1.30, 95% CI (−2.22, −0.38), *p* = 0.006]. The very high heterogeneity (*I^2^* = 95%) highlights the need to account for differences in disease activity assessment criteria across studies (Figure 4).

#### 3.1.8 BASFI

A total of 7 RCTs (12, 14, 17, 28–30, 35) including 571 patients were analyzed. The results demonstrated that TGP significantly improved BASFI scores compared with the control group [WMD = −2.06, 95% CI (−3.31, −0.81), *p* = 0.001; Figure 4].

#### 3.1.9 Schober

A total of 11 RCTs (12, 14–16, 19, 20, 22, 24, 26, 32, 33) involving 819 patients were included. Meta-analysis revealed no significant difference between TGP and control groups in improving Schober scores [WMD = −0.08, 95% CI (−0.40, 0.24), *p* = 0.64; Figure 4].

#### 3.1.10 Chest expansion

A total of 9 RCTs (12, 14–16, 19, 20, 22, 32) involving 589 patients were included. Meta-analysis showed that TGP significantly improved thoracic range of motion in AS patients [WMD = 0.27, 95% CI (0.17, 0.38), *p* < 0.00001], with excellent consistency across studies (*I^2^* = 0%; Figure 4).

#### 3.1.11 Adverse reactions

Adverse events were reported in 22 studies. Among these, one study documented adverse events exclusively in the control group (28), indicating high safety in the intervention group. Four studies reported lower adverse event rates in the TGP group than in the control group, though statistical significance was not specified (10, 15, 28, 34). Five studies found no statistically significant difference in adverse event incidence between groups (16, 18, 21, 31). The remaining studies demonstrated significantly lower adverse event rates in the TGP group (*p* < 0.05).

a Gastrointestinal reactions.

**Figure 5:**
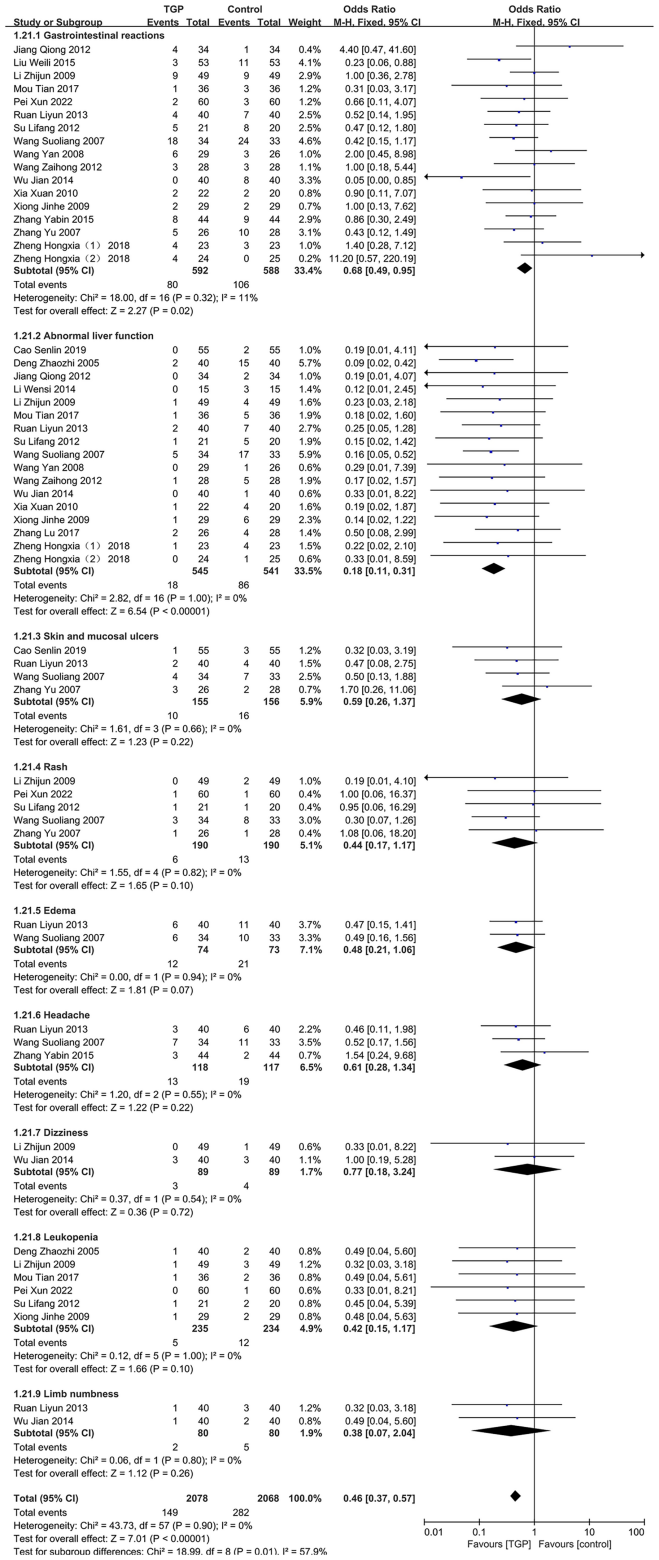
Forest plot of adverse reactions associated with AS.

A meta-analysis of 17 studies (14–17, 19–23, 26, 29–32, 34, 37) revealed that the incidence of gastrointestinal reactions was significantly lower in the TGP combination therapy group than in the control group (OR = 0.68, 95% CI [0.49, 0.95]; *I^2^* = 11%, *p* = 0.02; Figure 5).

b Abnormal liver function.

A meta-analysis of 17 studies (12, 14, 16, 17, 19–23, 26, 28, 29, 32–35) demonstrated that TGP combination therapy markedly reduced the incidence of abnormal liver function (OR = 0.18, 95% CI [0.11, 0.31]; *p* < 0.00001), with excellent consistency among studies (*I^2^* = 0%; Figure 5).

c Skin and mucosal ulcers.

Analysis of four studies (14, 15, 26, 35) showed no significant difference in the incidence of skin and mucosal ulcers between groups (OR = 0.59, 95% CI [0.26, 1.37]; *p* = 0.22; Figure 5).

d Rash.

A meta-analysis of five studies (14, 15, 17, 22, 37) demonstrated high consistency (*I^2^* = 0%), but no statistically significant difference in rash incidence between TGP combination therapy and control groups (OR = 0.44, 95% CI [0.17, 1.17]; *p* = 0.10; Figure 5).

e Edema.

Two studies (14, 26) reported no significant difference in edema incidence between the two groups (OR = 0.48, 95% CI [0.21, 1.06]; *I^2^* = 0%, *p* = 0.07; Figure 5).

f Headache.

Analysis of three included studies (14, 26, 31) indicated no significant difference in headache incidence between TGP combination therapy and control groups (OR = 0.61, 95% CI [0.28, 1.34]; *I^2^* = 0%, *p* = 0.22; Figure 5).

g Dizziness.

A meta-analysis of two studies (17, 29) indicated a trend toward a lower incidence of dizziness in the TGP combination therapy group compared with the control group, although the difference was not statistically significant (OR = 0.77, 95% CI [0.18, 3.24]; *p* = 0.72). Study consistency was high (*I^2^* = 0%; Figure 5).

h Leukopenia.

A meta-analysis of six studies (12, 17, 19, 22, 32, 37) found no significant difference in leukopenia incidence between the TGP combination therapy and control groups (OR = 0.42, 95% CI [0.15, 1.17]; *I^2^* = 34%, *p* = 0.10). Although high interstudy consistency was noted (*I^2^* = 0%), the pooled results did not reach statistical significance (Figure 5).

i Limb numbness.

Two meta-analyses (26, 29) demonstrated that while limb numbness showed a decreasing trend in the TGP combination therapy group (OR = 0.38, 95% CI [0.07, 2.04]), the difference was not statistically significant (*p* = 0.26; Figure 5).

#### 3.1.12 Evidence quality assessment

The GRADE evaluation revealed that among 18 meta-analytic outcomes, 11 provided moderate-quality evidence and 7 yielded low-quality evidence. The main reasons for downgrading included lack of risk-of-bias assessment and moderate-to-high heterogeneity, among other methodological limitations. Detailed quality ratings are presented in Table 2.

**Table 2:** GRADE evidence summary.

Outcome indicator	RCT	Sample size		Downgrade quality of evidence					Quality of evidence
		T	C	Risk of bias	Inconsistency	Indirectness	Imprecision	Publication bias	
TCM efficacy rate	13	477	453	serious	no	no	no	undetected	moderate
VAS	8	283	286	serious	serious	no	no	undetected	low
Morning stiffness duratio	16	615	613	serious	serious	no	no	undetected	low
ESR	22	858	856	serious	serious	no	no	undetected	low
CRP	20	743	739	serious	serious	no	no	undetected	low
BASDAI	7	265	264	serious	serious	no	no	undetected	low
BASFI	7	286	285	serious	serious	no	no	undetected	low
Schober	11	412	407	serious	serious	no	no	undetected	low
Chest expansion	9	297	292	serious	no	no	no	undetected	moderate
Gastrointestinal reactions	17	592	588	serious	no	no	no	undetected	moderate
Abnormal liver function	17	545	541	serious	no	no	no	undetected	moderate
Skin and mucosal ulcers	4	155	156	serious	no	no	no	undetected	moderate
Rash	5	190	190	serious	no	no	no	undetected	moderate
Edema	2	74	73	serious	no	no	no	undetected	moderate
Headache	3	118	117	serious	no	no	no	undetected	moderate
Dizziness	2	89	89	serious	no	no	no	undetected	moderate
Leukopenia	6	235	234	serious	no	no	no	undetected	moderate
Limb numbness	2	80	80	serious	no	no	no	undetected	moderate

### 3.2 Network pharmacology results

#### 3.2.1 Screening results of active components in white peony root

Screening of the active compounds in white peony root using the TCMSP database identified five components meeting the pharmacokinetic thresholds of OB > 30% and DL > 0.18. Compound identities and chemical structures were verified through the PubChem database. Except for hydroxypaeoniflorin, four compounds had confirmed structures, resulting in four final active components (Table 3).

**Table 3:** Active ingredients of white peony root.

No.	Name	Molecular formula
1	paeoniflorin	C_23_H_28_O_11_
2	hydroxy-paeoniflorin	-
3	paeonin	C_28_H_33_ClO_16_
4	albiflorin	C_23_H_28_O_11_
5	benzoylpaeoniflorin	C_30_H_32_O_12_

#### 3.2.2 TGP-target intersection for AS

**Figure 6:**
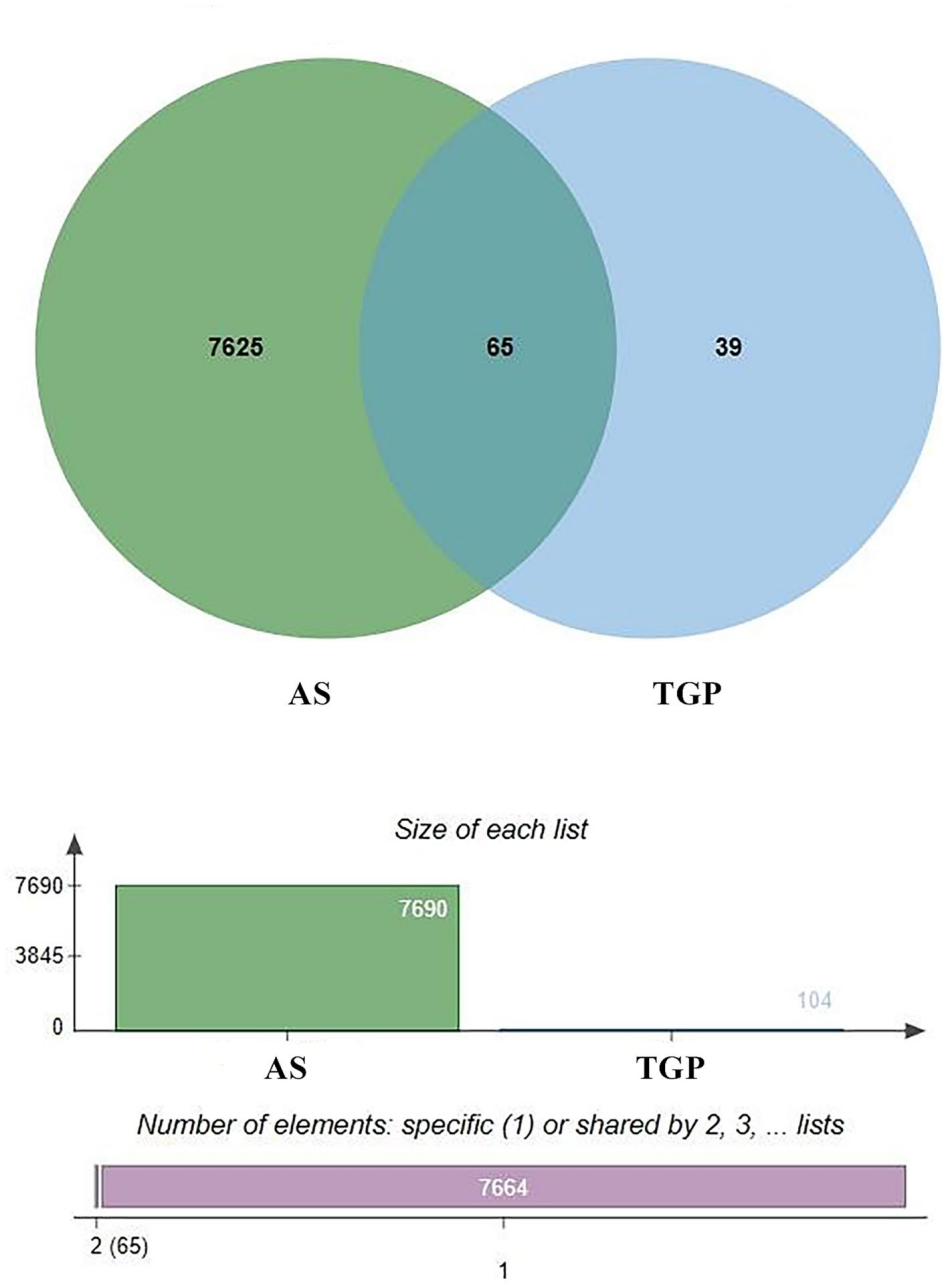
TGP and AS Venn diagram.

Prediction of molecular targets for these four active components in the SwissTargetPrediction database, followed by deduplication, yielded 104 credible target molecules. Searching the GeneCards database with the keyword “ankylosing spondylitis” identified 7,690 disease-related targets. Intersection analysis between TGP and disease targets produced 65 overlapping genes, visualized via a Venn diagram (Figure 6).

**Figure 7:**
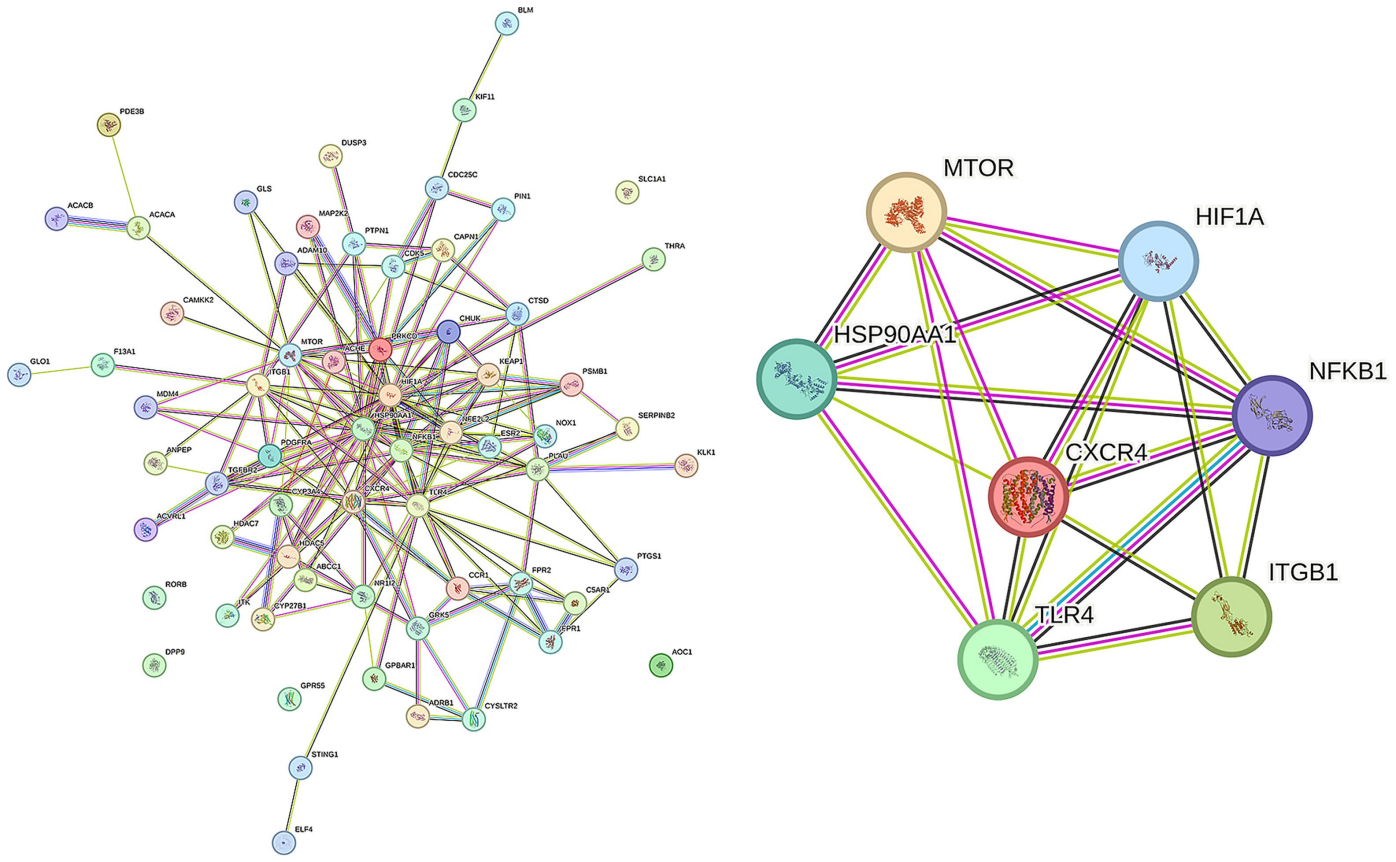
PPI network and analysis of TGP and AS potential targets.

To elucidate protein-level mechanisms of TGP action in ankylosing spondylitis (AS), the 65 intersecting genes were imported into the STRING 12.0 database for protein–protein interaction (PPI) analysis. Visualization with Cytoscape 3.7.1 produced the PPI network shown in Figure 7. Network topology analysis identified seven highly connected hub proteins—TLR4, NFKB1, HSP90AA1, MTOR, HIF1A, ITGB1, and CXCR4—representing core targets in the TGP–AS interaction network (Figure 7).

#### 3.2.3 GO and KEGG enrichment analysis

**Figure 8:**
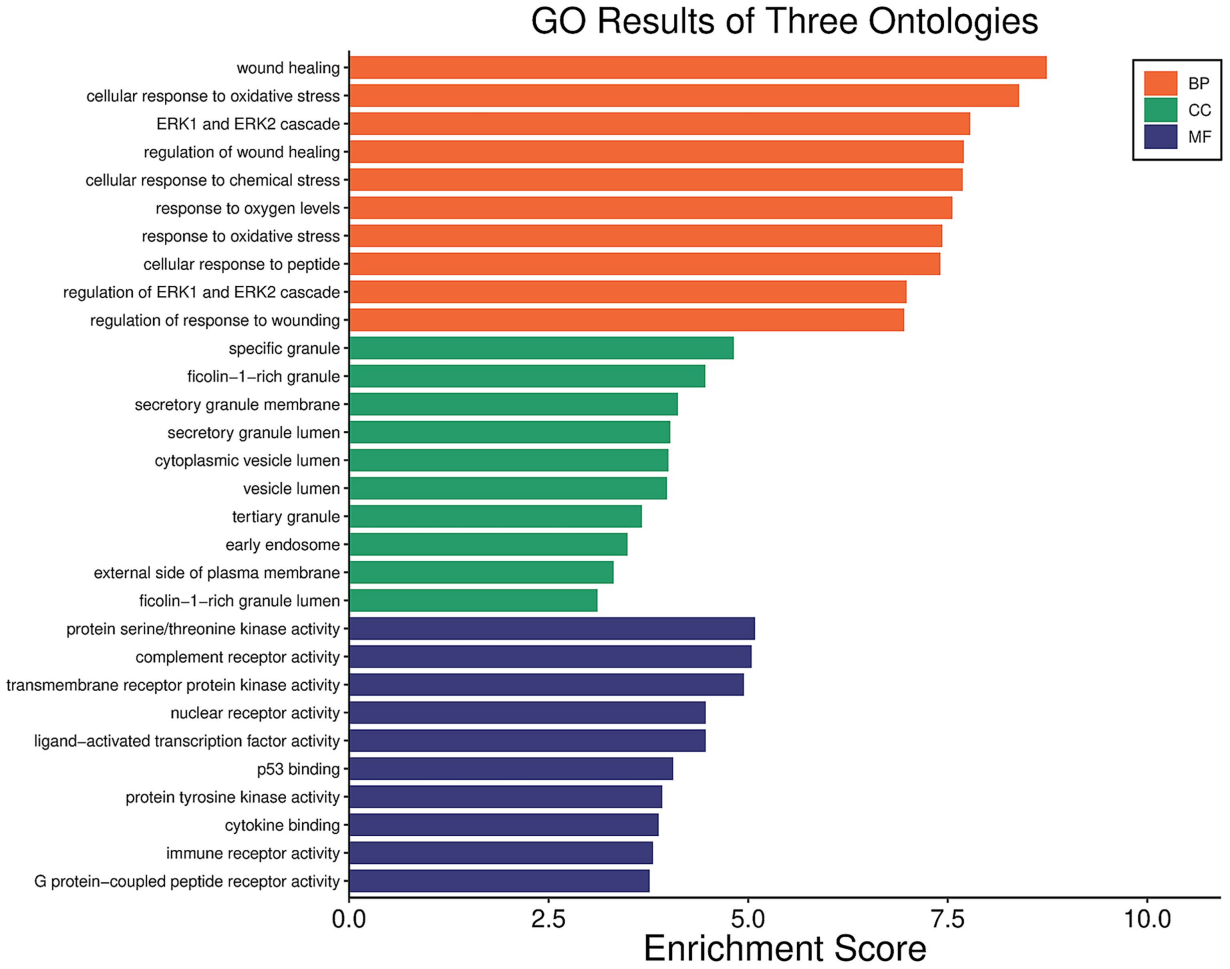
Bar chart of GO enrichment analysis for key targets of TGP-AS.

**Figure 9:**
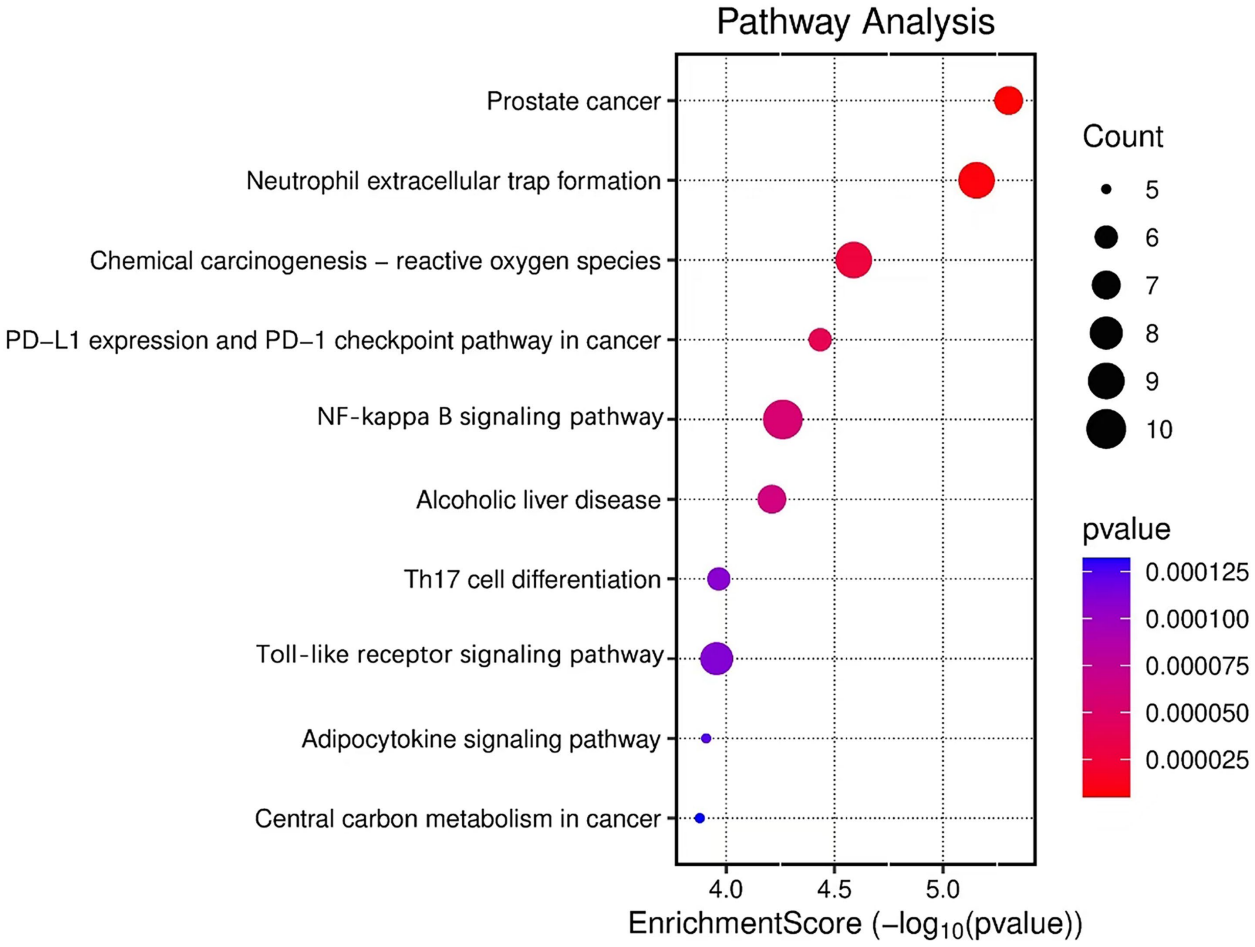
KEGG enrichment analysis of key targets in TGP-AS mulberry bubble chart.

To further clarify the core mechanisms of white peony root in AS treatment, functional enrichment and pathway analyses were conducted using the DAVID database for the intersecting targets. The top 10 targets were selected for detailed investigation. Gene Ontology (GO) analysis identified 1,109 significant terms (*p* < 0.05), comprising 949 in biological processes (BP), 59 in cellular components (CC), and 101 in molecular functions (MF). The top 10 GO terms, ranked by *p*-value, are presented in Figure 8. KEGG pathway enrichment revealed 84 significantly enriched signaling pathways (*p* < 0.05), with the top 10 analyzed in depth. The affected pathways were primarily related to cancer, Toll-like receptor signaling, and NF-κB signaling (Figure 9). These findings suggest that white peony root may modulate AS pathophysiology by regulating these critical signaling cascades.

#### 3.2.4 Molecular docking simulation of TGP and AS binding to major disease targets

**Figure 10:**
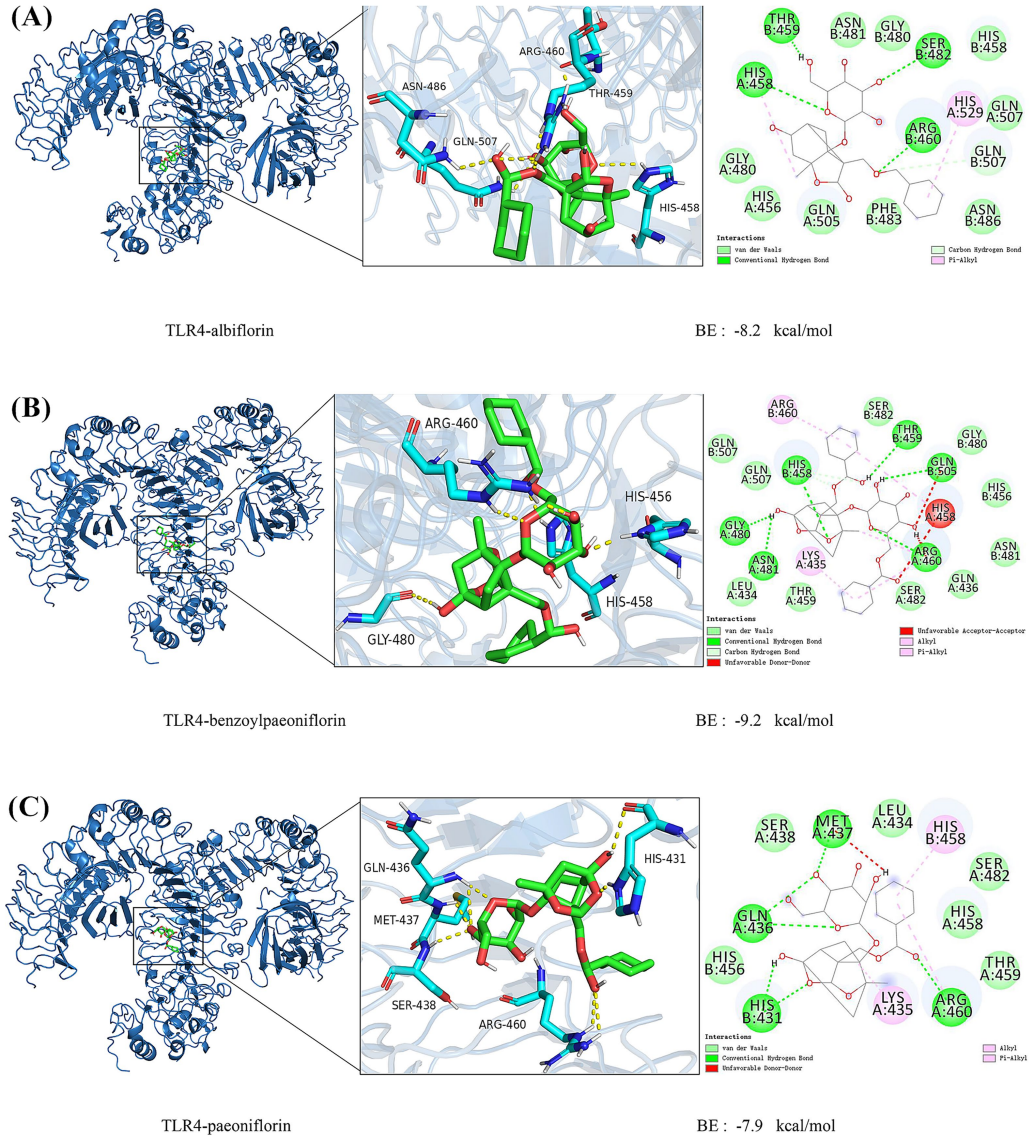
Molecular docking results of albiflorin with TLR4: **(A)** Molecular docking results of albiflorin with TLR4, **(B)** benzoylpaeoniflorin with TLR4, **(C)** paeoniflorin with TLR4.

Using both automated search (Automatic) and original ligand-based (Ligand) docking approaches, active site pockets were identified. Molecular docking was performed, with success indicated by T_Score parameters. Each small molecule generated 20 docking conformations under default conditions (Table 4). Docking analysis revealed that benzoylpaeoniflorin, paeoniflorin, and albiflorin exhibited favorable binding affinities with TLR4 (binding energies < −7.0 kcal/mol; Figures 10A–C).

**Table 4:** Molecular docking results.

No.	Name	TLR4 (PDBID 4g8a)
1	benzoylpaeoniflorin	7.1284
2	paeoniflorin	6.5466
3	albiflorin	5.429
4	paeonin	2.1803

#### 3.2.5 Molecular dynamics simulation

**Figure 11:**
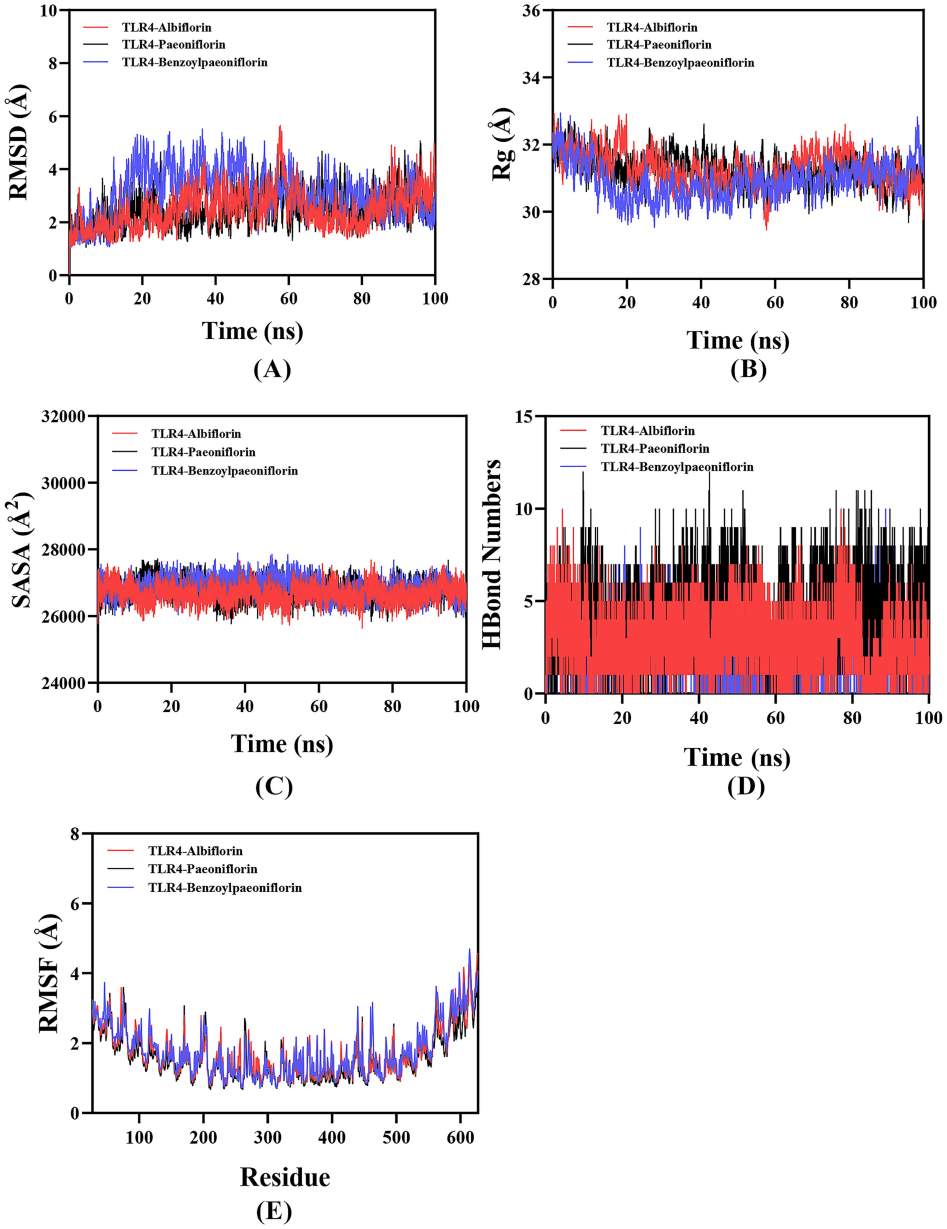
Molecular dynamics simulation of protein-ligand complexes. **(A)** RMSD values of the protein-ligand complex over time; **(B)** *R*g values of the protein-ligand complex over time; **(C)** SASA values of the protein-ligand complex over time; **(D)** Hbonds values of the protein-ligand complex over time; **(E)** RMSF values of the protein-ligand complex.

Root-mean-square deviation (RMSD) analysis was used to evaluate the conformational stability of protein–ligand complexes, quantifying deviations of atomic positions from their initial coordinates. Smaller RMSD values indicate greater structural stability. As shown in Figure 11A, the TLR4–Albiflorin, TLR4–Paeoniflorin, and TLR4–Benzoylpaeoniflorin complexes fluctuated during molecular dynamics simulations but stabilized around 3.3 Å, 2.5 Å, and 3.1 Å, respectively, confirming stable complex formation.

The radius of gyration (Rg) reflects changes in a protein’s overall conformation and serves as an indicator of structural compactness. The TLR4–Albiflorin, TLR4–Paeoniflorin, and TLR4–Benzoylpaeoniflorin complexes exhibited only minor Rg fluctuations throughout the simulation, suggesting limited conformational variation during motion (Figure 11B).

The solvent-accessible surface area (SASA) quantifies the extent of a protein’s surface exposed to solvent, thereby reflecting conformational and interaction-related changes. The simulation-derived SASA values for the three TLR4–ligand complexes (Figure 11C) displayed slight oscillations, indicating that small-molecule binding subtly modulates the protein’s microenvironment and induces modest alterations in solvent exposure.

Hydrogen bonding plays a pivotal role in maintaining the stability and specificity of ligand–protein interactions. As shown in Figure 11D, the TLR4–Albiflorin complex formed between 0 and 10 hydrogen bonds, with an average of approximately 7. The TLR4–Paeoniflorin complex exhibited 0–13 hydrogen bonds, also averaging around 7, while the TLR4–Benzoylpaeoniflorin complex maintained 0–10 hydrogen bonds, typically around 5. These results highlight that all three ligands engage in strong and stable hydrogen-bonding interactions with TLR4.

The root mean square fluctuation (RMSF) measures the flexibility of individual amino acid residues within a protein. As illustrated in Figure 11E, the RMSF values for all three TLR4–ligand complexes remained relatively low (predominantly < 4 Å), reflecting reduced residue mobility and enhanced overall complex stability.

## 4 Discussion

Epidemiological data indicate an average prevalence of 0.167% in Asia, accompanied by a notably high disability rate (38). AS has an insidious onset, with atypical early symptoms often resulting in misdiagnosis or delayed diagnosis. Limited public awareness further contributes to diagnostic delays, and many patients receive a definitive diagnosis only after spinal joint fusion has occurred, greatly complicating treatment. Consequently, the primary therapeutic goal in AS is early diagnosis and timely intervention to delay pathological new bone formation and improve long-term prognosis. Currently, pharmacologic treatments for AS include nonsteroidal anti-inflammatory drugs (NSAIDs), disease-modifying antirheumatic drugs (DMARDs), and biologics. However, long-term administration of these agents can cause adverse cardiovascular effects and elevate the risk of infections or malignancies. Moreover, NSAIDs—the first-line therapy—often exhibit limited efficacy in suppressing disease activity or preventing new bone formation. Traditional Chinese medicine has a long history of managing AS with a favorable safety profile. Total glucosides of paeony (TGP), extracted from *Paeonia lactiflora*, exhibit potent anti-inflammatory and immunomodulatory properties (39), effectively reducing inflammation and improving spinal mobility in AS patients. Despite its widespread clinical use in China, the molecular mechanisms underlying TGP’s therapeutic effects remain insufficiently understood and lack systematic, evidence-based validation. Therefore, this study aims to provide evidence-based support through meta-analysis and further explore TGP’s mechanistic pathways in AS using network pharmacology, molecular docking, and molecular dynamics simulations, thereby establishing a rigorous scientific foundation for its therapeutic application.

The meta-analysis included 28 studies encompassing 2,130 patients across China, providing strong statistical power and generalizability. Most studies described the generation of random sequences (e.g., using a random number table); however, the description of allocation concealment was often insufficient, which may introduce a certain risk of selection bias. Due to differences in the administration methods between TGP and control drugs in some studies, there were practical difficulties in blinding participants and personnel. Regarding attrition bias, the loss to follow-up rates in the included studies were generally low, suggesting that the risk of bias in this domain is relatively limited. In the revised Discussion section, we have also included a critical reflection on the quality assessment results: Owing to deficiencies in the blinding design of some studies, the incidence of patient-reported adverse reactions (e.g., subjective symptoms) in this meta-analysis may be overestimated or underestimated to some extent, and therefore these findings should be interpreted with caution. In contrast, the results for laboratory parameters and objective adverse events are relatively more reliable. In future research, allocation concealment should be optimized to the greatest extent possible, and a double-blind design should be adopted whenever feasible. If a double-blind approach is not possible due to ethical or practical considerations, blinding of outcome assessors should be strengthened to minimize measurement bias. The results demonstrated that TGP combination therapy significantly enhanced overall TCM efficacy, reduced disease activity (as indicated by decreases in BASDAI and VAS scores), mitigated inflammation (evidenced by lower ESR and CRP levels), and improved clinical manifestations (shorter morning stiffness duration, enhanced chest expansion, and decreased BASFI scores) compared to control treatments. Additionally, TGP therapy reduced the incidence of gastrointestinal reactions and hepatic dysfunction. In TCM theory, white peony root nourishes the blood, soothes the liver, and alleviates pain. Pharmacological studies corroborate these effects, showing that TGP possesses hepatoprotective properties, including the reduction of hepatic inflammatory infiltration and steatosis. These findings are consistent with clinical observations and provide mechanistic evidence supporting TGP’s role in AS treatment.

During the data analysis phase, we performed subgroup analyses based on pre-defined potential sources of clinical heterogeneity (such as patient baseline characteristics, treatment duration, and type of control). These analyses revealed that while effect sizes varied somewhat across subgroups, substantial heterogeneity persisted within most subgroups, suggesting that no single clinical or methodological factor could fully explain the observed variability. We also conducted sensitivity analyses by omitting one study at a time. The results showed that excluding any single study did not alter the direction of the pooled effect size, and although the heterogeneity statistic fluctuated slightly, it generally remained high. This suggests that the heterogeneity was not driven by a single outlier study but rather reflected more pervasive differences across the included studies. Based on these exploratory findings, we propose that the sources of the high heterogeneity are likely multi-dimensional and complex. Factors such as potential genetic polymorphisms, disease subtypes, or differences in baseline disease severity among patients, all of which could influence treatment effects, are often inadequately reported or standardized in the original studies and cannot be effectively stratified in conventional subgroup analyses. These micro-level differences represent important potential sources of heterogeneity. Furthermore, variations in the definition of control groups may have compounded these effects, potentially amplifying the between-study heterogeneity. Given the substantial unexplained heterogeneity in the current evidence base, the conclusions of this meta-analysis should be interpreted with caution. We recommend that future research prioritize well-designed, rigorously reported original studies, with particular emphasis on standardized outcome measurement and reporting, to facilitate more precise exploration of heterogeneity in subsequent analyses.

TGP is a traditional Chinese medicine extract originating from China, and its clinical application has been recognized by Chinese diagnostic and treatment guidelines for AS. Consequently, the existing body of evidence for TGP is inherently rooted in Chinese research practices. Given that this agent has not been extensively promoted or rigorously investigated outside of China, the current study’s exclusive focus on indigenous Chinese randomized controlled trials does not constitute deliberate selection bias. Furthermore, the homogeneity of the study populations, diagnostic criteria, and treatment protocols across the included trials enhances the internal validity of our analysis, minimizes confounding factors, and allows for more precise effect estimates. While this approach limits external validity, it establishes a robust and reliable foundation for future international research. Nonetheless, we believe that multi-ethnic, cross-national randomized controlled trials are still warranted to validate its efficacy in diverse populations.

Network pharmacology analysis identified four principal active components of TGP—paeoniflorin, paeonin, albiflorin, and benzoylpaeoniflorin—which share multiple overlapping molecular targets with AS. Protein–protein interaction (PPI) network and topological analyses revealed key targets including TLR4, NFKB1, HSP90AA1, HIF1A, MTOR, ITGB1, and CXCR4. AS primarily manifests as enthesitis, where mechanical stress at tendon–bone junctions induces mesenchymal and fibroblast activation. These cells express damage-associated molecular patterns (DAMPs) that stimulate the innate immune response via TLR4, recruiting Th17 cells and triggering inflammatory cascades (40). NF-κB, a downstream effector of TLR4, mediates the expression of pro-inflammatory cytokines and contributes to spinal and joint pain, swelling, and stiffness in AS (41). Animal models demonstrate markedly elevated TLR4 and NF-κB expression in AS compared to controls (42), confirming that the TLR4/NF-κB signaling axis serves as a canonical pathway regulating inflammation and a critical immune-related target for AS therapy (43). Multiple network pharmacology studies further highlight HSP90AA1 as a key molecular target (44, 45). The gene encodes heat shock protein HSP90*α*, which assists in protein folding, maintains structural stability, and facilitates refolding or degradation of misfolded proteins. Under stress conditions, aberrant HSP90α expression is observed in various tissues, and elevated levels may serve as prognostic biomarkers in certain diseases (46). Pathological new bone formation—the major cause of disability in AS—is closely linked to local hypoxia. HIF1A, a master regulator of cellular hypoxic responses, mediates autophagy activation and modulates bone metabolism (47). However, the specific roles of HSP90α and HIF1A in AS remain unexplored and represent promising directions for future research. Aberrant autophagy contributes to inflammatory pathogenesis in autoimmune disorders. The mTOR protein, a downstream effector of the PI3K/Akt/mTOR signaling pathway, regulates autophagy and influences cell growth, metabolism, and survival. Studies comparing mesenchymal stem cells (MSCs) from AS patients and healthy controls revealed significantly upregulated PI3K/AKT/mTOR pathway activity in AS-derived MSCs, accompanied by impaired autophagy and reduced anti-inflammatory capacity. Under TNF-α stimulation, hyperactivation of this pathway exacerbates chronic inflammation, suggesting that dysregulated autophagy plays a pivotal role in AS progression (48). Further investigation into key molecular mediators of this pathway is warranted. The role of ITGB1 in AS is not well characterized, though evidence suggests it may enhance osteoclast differentiation via lncRNA-mediated upregulation, contributing to pathological bone remodeling (49). Similarly, CXCR4—the receptor for chemokine CXCL12—activates downstream G protein-coupled signaling cascades, including PI3K/AKT, MAPK, and NF-κB pathways. These pathways collectively regulate lymphocyte migration, inflammatory cytokine secretion, and immune homeostasis (50). The precise mechanisms of CXCR4 in AS, however, remain to be elucidated through further study.

GO and KEGG enrichment analyses revealed that the Toll-like receptor (TLR) and NF-κB signaling pathways play pivotal roles in TGP-mediated regulation of ankylosing spondylitis (AS). Toll-like receptors, key components of the innate immune system, are responsible for pathogen recognition and activation of adaptive immune responses—an insight that earned the 2011 Nobel Prize in Physiology or Medicine for elucidating the first step in the human immune response. Upon ligand recognition, TLRs initiate intracellular signaling cascades that promote the synthesis and release of inflammatory and adhesion molecules, while also modulating the differentiation and function of Th17 and Treg cells. These processes collectively intensify the inflammatory milieu characteristic of AS (51, 52). Peripheral blood mononuclear cell (PBMC) analysis from AS patients demonstrated significantly elevated TLR4 expression, and regression analysis identified high TLR4 mRNA levels as an independent risk factor for AS (53). Increasing evidence suggests a strong link between inflammation and pathological new bone formation in AS. Specifically, elevated IL-17A promotes TLR3^+^ MSC2 polarization, enhancing osteogenic differentiation through activation of the Wnt10b/RUNX2 signaling pathway, thereby driving aberrant bone formation (54). Collectively, these findings indicate that TLRs not only mediate inflammatory responses in AS but also regulate bone metabolism pathways that lead to ligamentous ossification. Thus, targeting the TLR pathway may represent a promising therapeutic strategy for AS. In resting cells, NF-κB remains sequestered in the cytoplasm by the inhibitory protein IκB. Upon stimulation, IκB undergoes phosphorylation and degradation, liberating NF-κB to translocate into the nucleus, where it activates transcription of genes involved in immune regulation, inflammation, cell proliferation, and apoptosis. Recent studies highlight the central role of NF-κB signaling in AS pathogenesis. As a master transcription factor for inflammation (55), NF-κB induces the expression of numerous cytokines that amplify inflammatory cascades in AS. Furthermore, beyond its inflammatory functions, the NF-κB pathway facilitates M1 macrophage polarization and osteoclast differentiation. Pharmacological inhibition of this pathway has been shown to mitigate both inflammation and bone destruction in AS patients (56). Accordingly, TGP may exert therapeutic efficacy in AS by modulating multiple cytokine-mediated signaling pathways, including TLR and NF-κB cascades.

**Figure 12:**
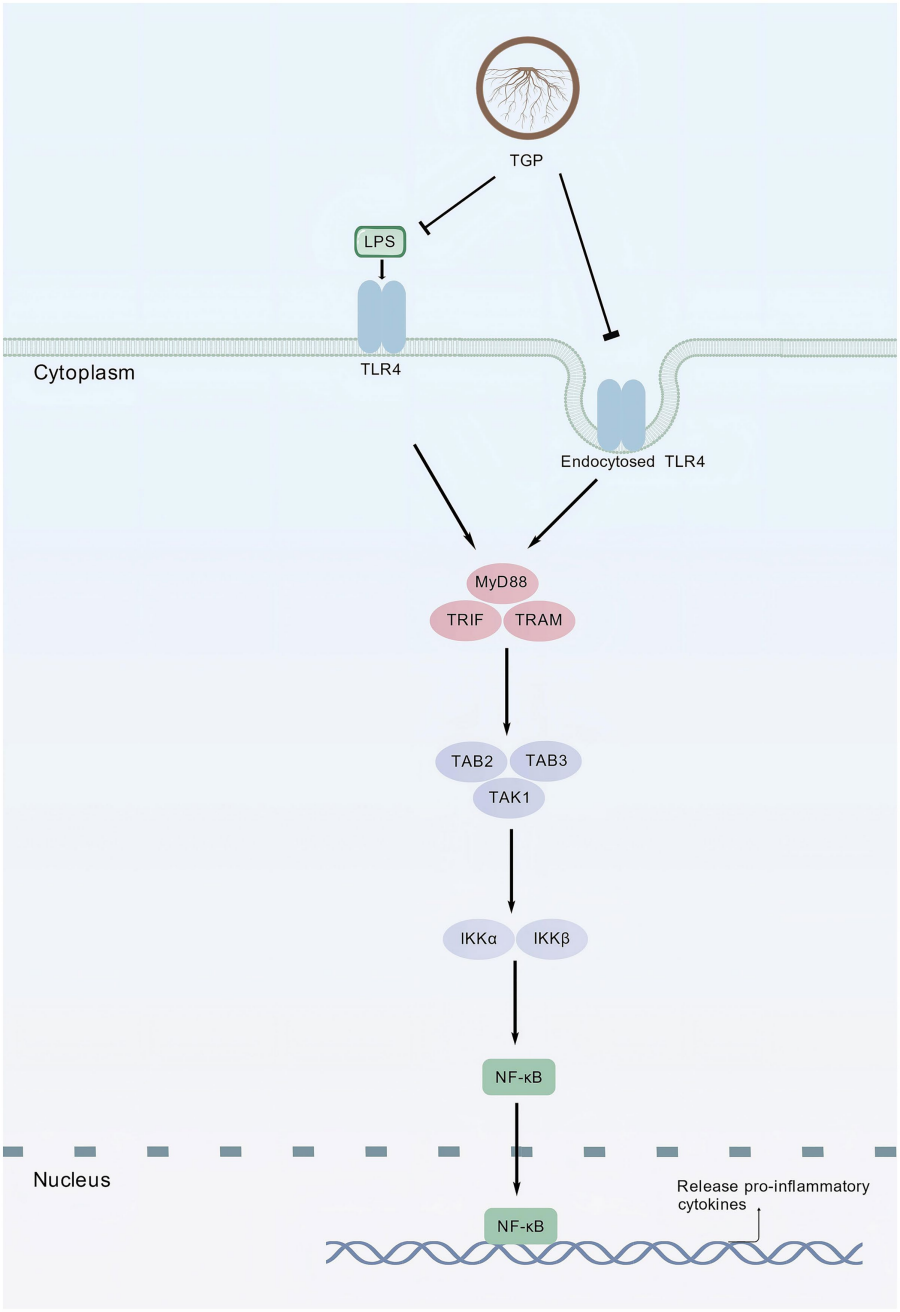
Potential mechanism of TGP for AS (created using BioGDP.com).

Molecular docking analyses revealed that benzoylpaeoniflorin, paeoniflorin, and albiflorin—key active components of TGP—exhibit strong binding affinities toward TLR4, with benzoylpaeoniflorin showing the highest affinity (binding energy: −9.2 kcal/mol). Subsequent molecular dynamics simulations confirmed the conformational stability of TLR4–Albiflorin, TLR4–Paeoniflorin, and TLR4–Benzoylpaeoniflorin complexes. These findings suggest that TGP may exert its therapeutic effects in AS, at least in part, through direct interaction with TLR4 (Figure 12) (57). Overall, TGP appears to regulate AS pathophysiology via a multi-target, multi-pathway mechanism involving both immune and bone metabolism modulation.

Due to current technical limitations and the lack of a well-established classical animal model for AS to verify target specificity and the associated signaling pathways of TGP in AS treatment, only molecular dynamics simulations were employed in this study. In the future, our research team will develop disease models that more closely mimic AS to validate our scientific findings. This will not only help elucidate the biological mechanisms underlying the differential therapeutic effects observed in this study but also provide a scientific basis for identifying potential patient populations that may benefit most, thereby facilitating personalized treatment strategies. Moreover, validation using modern bioinformatics approaches or Mendelian randomization will offer stronger theoretical support for the clinical application of TGP.

## 5 Conclusion

Compared with conventional treatments, TGP combination therapy markedly improves spinal function and quality of life in AS patients, with pronounced anti-inflammatory and immunomodulatory effects and favorable safety. Owing to its hepatoprotective properties, TGP reduces the incidence of liver function abnormalities relative to traditional therapies. This study further identified potential molecular targets for TGP in AS, including TLR4, NF-κB1, HSP90AA1, HIF1A, mTOR, ITGB1, and CXCR4—proteins primarily involved in inflammation suppression and bone metabolism regulation. Collectively, these findings reinforce that TGP’s therapeutic actions are mediated through modulation of the TLR and NF-κB pathways. Molecular docking and dynamics results underscore benzoylpaeoniflorin’s superior binding affinity, further supporting TLR4 as a key molecular target in TGP-mediated AS treatment.

Despite these promising findings, this study has several limitations. First, most RCTs included in the meta-analysis were conducted in China, potentially introducing racial and regional biases that may limit generalizability. Future research should include larger, high-quality, multicenter RCTs across diverse populations to validate these results. Second, although comprehensive searches were performed, the literature review was restricted to Chinese and English databases, which may have led to the omission of relevant studies published in other languages. Finally, due to constraints in study design and manuscript length, this research did not experimentally validate predictions derived from network pharmacology, molecular docking, and molecular dynamics simulations. Future studies will incorporate *in vitro* and *in vivo* experiments to substantiate TGP’s therapeutic mechanisms and efficacy in AS.

## Data Availability

The original contributions presented in the study are included in the article/supplementary material, further inquiries can be directed to the corresponding author.

## Footnotes

1
https://old.tcmsp-e.com/

2
https://www.pharmgkb.org/

3
https://cn.string-db.org/

4
http://pubchem.ncbi.nlm.nih.gov/

5
http://www.rcsb.org/
